# Effect of tricalcium silicate-based intracanal dressing on bone resorption and inflammatory mediators in periapical lesions: a randomized controlled clinical trial

**DOI:** 10.1007/s10266-025-01093-6

**Published:** 2025-03-28

**Authors:** Oğuzhan Ünal, Meltem Sümbüllü, Esra Laloğlu

**Affiliations:** 1 Department of Endodontics, Faculty of Dentistry, Karabük University, Karabük, Türkiye; 2 Department of Endodontics, Faculty of Dentistry, Ataturk University, Erzurum, Türkiye; 3 Department of Medical Biochemistry, Faculty of Medicine, Atatürk University, Erzurum, Türkiye

**Keywords:** Bone resorption, Inflammatory mediators, Intracanal dressing, Periapical lesion, Tricalcium silicate-based intracanal dressing

## Abstract

This study aimed to investigate the effect of tricalcium silicate- and calcium hydroxide-based intracanal dressing on the release of nuclear factor kappa B ligand (RANKL), osteoprotegerin (OPG), tumor necrosis factor-alpha (TNF-α), prostaglandin-E2 (PGE-2), and transforming growth factor-beta (TGF-β) in asymptomatic periapical lesions. The 60 patients included in the study were randomly divided into two groups according to the intracanal dressing. After removing gutta-percha from the root canals, RANKL, OPG, TNF-α, PGE-2, and TGF-β samples were obtained from the apical tissues using three paper cones and the selected dressing material was placed into the canals. In the second appointment, the dressing was removed, and the second samples were taken using the same method. Pre-treatment and post-treatment RANKL, OPG, TNF-α, PGE-2, and TGF-β levels were determined using an enzyme-linked immunosorbent assay test. Data were analysed using *t*-test, the Mann–Whitney *U* test, the Wilcoxon test, The Fisher–Freeman–Halton test, and Path analysis. No significant difference was found between the groups in terms of demographic variables (age, gender, tooth region, and smoking) (*p* > 0.05). There was a statistically significant decrease in RANKL/OPG, TNF-α, PGE-2, and TGF-β levels before and after treatment in both groups (*p* < 0.05). A significant difference was observed in the percentage change of RANKL/OPG, TNF-α, and TGF-β ratios between the groups (*p* < 0.05). A statistically higher decrease in RANKL/OPG, TNF-α, and TGF-β levels was observed in the calcium hydroxide-based intracanal dressing group (*p* < 0.05). No significant difference was observed between the groups in terms of PGE-2 percentage change (*p* > 0.05). Tricalcium silicate-based intracanal dressing effectively reduced RANKL/OPG, TNF-α, PGE-2, and TGF-β levels in periapical lesions, but calcium hydroxide-based intracanal dressing resulted in a higher percentage reduction in RANKL/OPG, TNF-α, and TGF-β levels. The effects of both medicaments on PGE-2 levels were similar.

**Trial registration** The study was registered in clinical trials database on 12 March 2024 (https://www.clinicaltrials.gov). Registration number: NCT06307678.

## Introduction

Apical periodontitis is an inflammatory disease caused by the infection of the tissues surrounding the alveolar bone [1]. This condition is usually the result of an infection caused by bacteria and often leads to periapical bone resorption [2]. The resulting infection or inflammation leads to the release of various inflammatory mediators as a response to the immune system. These mediators include cytokines, chemokines, and neuropeptides, which are secreted by innate and acquired immune cells. As inflammation progresses, these inflammatory mediators diffuse into the periapical region [3]. This can lead to the development of apical osteolytic lesions and asymptomatic apical periodontitis due to widening of the periodontal ligament space or bone resorption. Osteolytic lesions are mainly caused by activated osteoclasts. Various cytokines, such as interleukin-1 (IL-1) and tumor necrosis factor-alpha (TNF-α), trigger the differentiation and activation of osteoclast progenitor cells through the osteoprotegerin (OPG)/nuclear factor kappa B ligand (RANKL)/nuclear factor kappa B (RANK) complex [4]. RANKL levels are increased by proinflammatory cytokines, such as IL-1β, IL-6, IL-11, and TNF-α, while cytokines that promote the production of OPG include TGF-α, TGF-β, IL-1α, and IL-18 [5]. The immune response, including the production of TNF-α and PGE2, has an important effect on the maintenance of the inflammatory process, destruction of bone tissue and remodeling of periapical lesions [6].

A combination of mechanical instrumentation and irrigation solutions is preferred to remove microorganisms from the root canal system, remove organic and inorganic debris, eliminate the smear layer [7]. However, several studies have shown that antibacterial irrigation with mechanical instrumentation can clear only 50–70% of infected root canals of microorganisms. [1]. For this reason, dressing materials are used between appointments, with the expectation of enhancing the prognosis of endodontic treatment [8, 9].

It has been demonstrated that calcium hydroxide, by dissociating into calcium and hydroxyl ions in a hydrophilic environment, promotes mineralization and provides an alkaline environment [10]. However, prolonged exposure to calcium hydroxide-based intracanal dressings has been suggested to lead to collagen degradation, thereby weakening the root dentin [11].

Tricalcium silicate-based intracanal dressing was inspired by the success of tricalcium silicate-based material such as MTA (Mineral Trioxide Aggregate), a type of hydraulic cement used in endodontic treatments [12]. It regulates the functions of host cells involved in bone repair and supports the survival and differentiation of osteoblasts, which are actively involved in periapical healing, allowing the formation of mineralized tissue [13]. Tricalcium silicate-based intracanal dressing (Bio-C Temp, Angelus, Brazil) is indicated in endodontic treatments as an intracanal dressing, as well as in apexification and regeneration treatments [14]. There are in vitro studies on the osteogenic and bioactive potential of Bio-C Temp [15, 16], but there are no clinical studies examining the effect of tricalcium silicate based intracanal dressing on bone destruction and the release of inflammation mediators in teeth with apical periodontitis.

This study aimed to investigate the effect of tricalcium silicate (Bio C Temp), and calcium hydroxide (Calcicur, Voco GmbH, Cuxhaven, Germany)-based intracanal dressings on the release of RANKL/OPG, TNF-α, PGE-2 and TGF-β in periapical lesions in nonsurgical endodontic retreatment of single-rooted teeth with asymptomatic apical periodontitis. The null hypothesis was that there would be no difference in the the release of RANKL/OPG, TNF-α, PGE-2 and TGF-β in periapical lesions between the tricalcium silicate- and calcium hydroxide- based intracanal dressings.

## Materials and methods

### Patient selection

This randomized, single-blind clinical trial was conducted according to the PRIRATE 2020 (Preferred Reporting Items for Randomised Trials in Endodontics 2020) Guidelines. The ethics committee approval was obtained from the local university clinical research ethical committee with the decision numbered B.30.2.ATA.0.01.00/1 and registered in the clinical trials database (www.clinicaltrials.gov) (NCT06307678).

The sample size was calculated using the G*Power program (Franz Faul, University of Kiel, Germany) based on data from a previous study that evaluated mediator levels in periapical lesions [17]. It was determined that at least 20 patients should be included per group (*α* = 0.05, power = 0.95, effect size = 1.0612). To enhance statistical power and account for possible dropouts, each group was determined to consist of 30 patients. All the participants fully understood the purpose of the study and signed informed consent forms.

The patients ranged in age from 18 to 59 years. Incisor, canine, and premolar teeth that had previously undergone root canal treatment, exhibited no pain or swelling, showed negative responses to palpation and percussion, and had a periapical index (PAI) score of > 2 with a diagnosis of asymptomatic apical periodontitis were included in the study. Periapical radiographs taken from different angles were used to assess the periapical status and root canal anatomy. To prevent difficulties in preparation, obturation, and restoration in molar teeth and to standardize treatments, only teeth with a single root canal were included in the study.

Patients classified as ASA II or higher, pregnant women, and those with a Schilder canal curvature exceeding 25° or internal/externsl root resorption were excluded. Additionally, patients with generalized periodontitis, or periodontal pockets greater than 3 mm, as well as those who had used NSAIDs within the past 24 h or antibiotics within the last 3 months, were not included.

### Study protocol

The randomization procedure was carried out using the online platform www.randomizer.org, which generates random numbers for assigning participants to experimental groups. Patient allocation followed the sequential order determined by this tool. The randomization process and its implementation were managed by the secretary.

The teeth were isolated with a rubber dam, the crown and surrounding structures were disinfected with 30% H_2_O_2_ for 30 s and 2.5% NaOCl was applied for the same period. Subsequently, 5% sodium thiosulfate was used to deactivate the effect of the NaOCl. Traditional access openings were prepared following complete caries excavation and removal of any previous restoration. The filling material from the cervical third was removed using Gates Glidden burs. The working length was determined using an electronic apex locator (Propex Pixi, Dentsply Sirona, Ballaigues, Switzerland) and a 15 K file (Mani Inc.; Utsunomiya, Tochigi, Japan). The length was established as soon as the “over” signal was observed on the apex locator, and the procedure was performed 0.5 mm short of this length. The working length was then confirmed using radiography.

Root canal shaping was completed using R25 and R50 Reciproc files (VDW, Munich, Germany) at the working lengths to finalize the canal preparation. The R25 file (VDW) was used to remove the root canal filling material, while the R50 file (VDW) was used to complete the root canal shaping. After each file was used, the root canals were irrigated with 2 ml of 1% NaOCl. Before obtaining the samples, the canals were irrigated with 5 ml each of 1% NaOCl, neutralized with 0.5% sodium thiosulphate, and then irrigated with distilled water, with each step lasting for 1 min. A sterile paper point (size #20; Dentsply Maillefer, Ballaigues, Switzerland) was placed into the root canals beyond the 2 mm root apex and were kept in this area for 1 min.

The procedure was repeated with three paper cones. Paper cones were cut 4 mm from the tip and the samples was stored in Eppendorf tubes containing a phosphate buffer saline at − 80 °C.

Afterward, the root canals were dried with paper points, and two groups were planned according to the intracanal treatment group. Calcium hydroxide based intracanal dressing (Calcicur) or tricalcium silicate based intracanal dressing (Bio-C Temp) was placed in the canal, 1 mm shorter than the working length using the applicator tip.

**Fig. 1:**
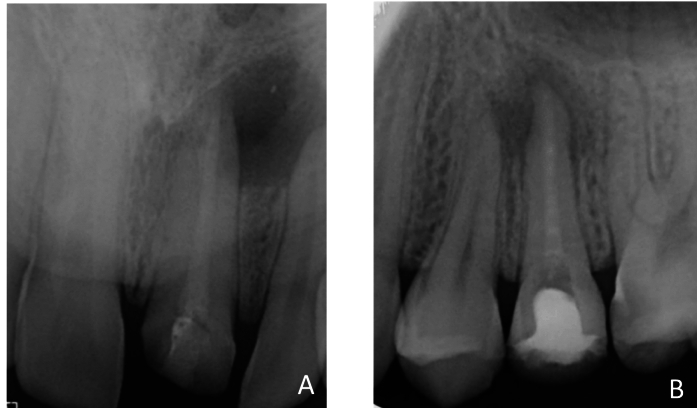
Periapical radiographs were taken in patients to assess the placement of intracanal dressing material. **A** Calcium silicate-based intracanal dressing, **B** calcium hydroxide-based intracanal dressing

The canal orifices were temporarily closed using a sterile teflon pallet and the coronal cavities were restored with Cavit G (3M ESPE, Seefeld, Germany). Radiographs were taken to confirm the presence of the dressing placed in the canal (Fig. 1).

Seven days later, the root canals were accessed aseptically under rubber dam isolation using the disinfection protocol as previously described. The medication was mechanically removed using a master apical file and irrigated with 5 ml of distilled water. Then, the root canals then filled with 5 ml of 17% EDTA (Endo-Solution; Cerkamed, Wojciech, Poland) and the solution was activated using the EndoActivator (Dentsply Tulsa Dental Specialties, Tulsa, OK). Finally, the canals were irrigated again with 5 ml of saline solution. The final samples were collected from the apical tissue as previously described and stored at − 80 °C. The root canals were filled with cold lateral condensation technique using guta percha cones and sealer (Sealapex, Sybron Kerr, Brea, CA).

### Determination of RANKL, OPG and inflammatory mediators levels using enzyme-linked immunosorbent assay (ELISA)

The RANKL, OPG, TNF-α, TGF-β, and PGE2 levels in the samples taken from the interstitial fluid were measured on the same day to avoid inter-day variation. The serum PGE2, RANKL, OPG, TNF-α, and TGF-β levels were measured through the ELISA method using a human PGE2 ELISA kit (Sunred, China), a human RANKL ELISA kit (Sunred, China), a human OPG ELISA kit (Sunred, China), a human TNF-α ELISA kit (Sunred, China), and a human TGF-β ELISA kit (Sunred, China) according to the manufacturer’s instructions. Eppendorf tubes stored at − 80 °C were thawed gradually 1 day before the study. The samples collected on the study day were vortexed, the tubes were placed in the centrifuge, and the paper points were collected at the bottom. Briefly, the procedure applied for measurement is as follows: in 96-well microplates coated with specific monoclonal antibodies against human PGE2, RANKL, OPG, TNF-α, and TGF-β, serum and standard solutions obtained using serial dilutions at decreasing concentrations were added. The PGE2, RANKL, OPG, TNF-α, and TGF-β molecules in the samples were bound to these coated antibodies. The unbound molecules were removed by washing. A second antibody specific for PGE2, RANKL, OPG, TNF-α, and TGF-β and labelled with biotin was added to the wells. After another wash, the peroxidase enzyme bound with streptavidin was added. The peroxidase enzyme in this complex bound with avidin oxidized the 3,3′ 5,5'-tetra-methyl benzidine added to the medium and caused a color change in direct proportion to the concentration of PGE2, RANKL, OPG, TNF-α, and TGF-β in the samples. Acid was then added to each well to stop the reaction. The absorbance values of each well were measured with a spectrophotometer at a wavelength of 450 nm. The PGE2, RANKL, OPG, and TNF-α concentrations in each sample were calculated in ng/L and the TGF-β concentration in ng/ml from the absorbance–concentration graph using standards prepared at decreasing concentrations, and their levels were evaluated using an ELISA reader.

### Statistical analysis

The data were analyzed using IBM SPSS v23 and IBM AMOS v24 (SPPS Inc, Chicago, IL, USA). Compliance with normal distribution was analyzed using the Shapiro–Wilk test and the multivariate normality assumption. The Mann–Whitney *U* test was used to compare non-normally distributed data, and independent two-sample *t* test was used to compare normally distributed data. In the comparison of non-normally distributed data over time within groups, the Wilcoxon test was used, while the paired two-sample t test was used to compare normally distributed data.

The Fisher–Freeman–Halton test and Yates correction were used to compare the categorical variables by groups. Path analysis was used to analyze the effect of the independent variables on the percentage changes, and the maximum likelihood method was used as the calculation method. The results of the analyses were presented as the mean ± standard deviation, and the median (minimum–maximum) was presented for the quantitative data. The significance level was set at 5% (*p* < 0.05).

## Results

**Fig. 2:**
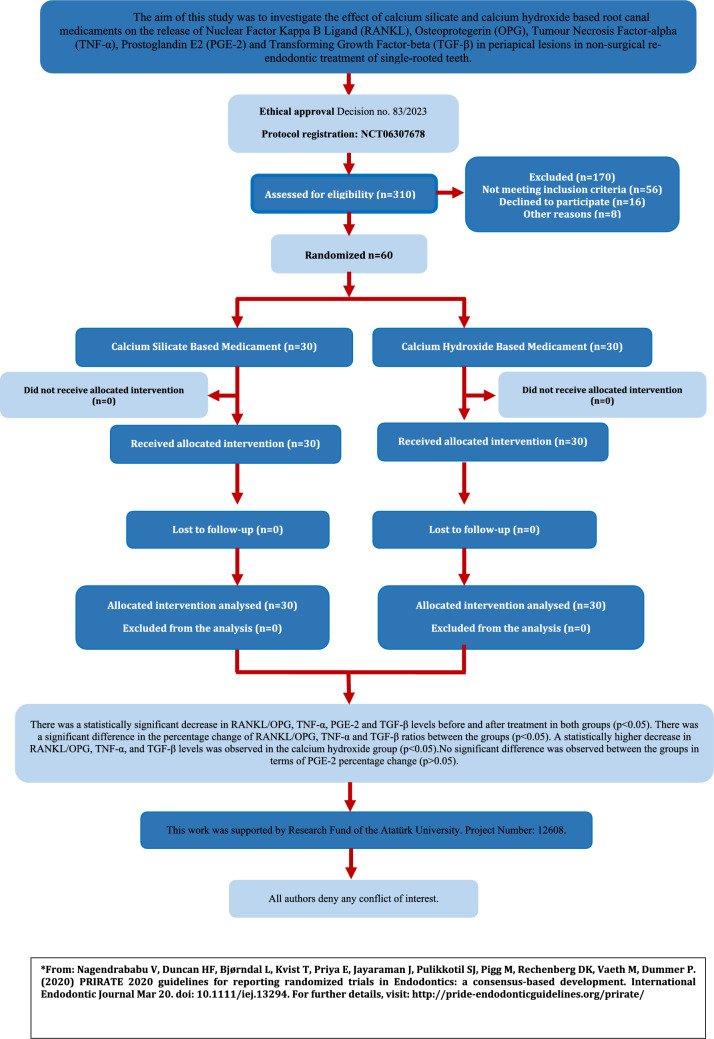
PRIRATE 2020 flowchart

The samples were taken from a total of 60 patients at the Faculty of Dentistry, Atatürk University. There was no loss of patients or follow-up in the calcium hydroxide and tricalcium silicate-based root canal dressing groups. Details about the inclusion process of the participants are shown in Fig.  2. There was no statistically significant difference between the groups in terms of age, gender, tooth region and smoking habits (*p* > 0.05) (Table 1).

**Table 1:** Comparison of demographic characteristics by groups

	Calcium silicate	Calcium hydroxide	Total	Test statistics	*p* value
*Age*					
18–29	10 (33.3)	16 (53.3)	26 (43.3)	2.539	0.470**
30–39	8 (26.7)	5 (16.7)	13 (21.7)		
40–49	8 (26.7)	6 (20)	14 (23.3)		
50–59	4 (13.3)	3 (10)	7 (11.7)		
*Gender*					
Female	21 (70)	16 (53.3)	37 (61.7)	1.128	0.288*
Male	9 (30)	14 (46.7)	23 (38.3)		
*Tooth region*					
Maxillary tooth	15 (50)	16 (53.3)	31 (51.7)	0.000	1.000*
Mandibular tooth	15 (50)	14 (46.7)	29 (48.3)		
*Smoking habit*					
No	18 (60)	16 (53.3)	34 (56.7)	0.068	0.794*
Yes	12 (40)	14 (46,7)	26 (43,3)		

The distribution of patients evaluated by age, gender, dental region, and smoking habit is shown. There was no statistically significant difference among the groups regarding age, gender, dental region, and smoking status (*p* > 0.05)

*Yates correction

** Fisher–Freeman–Halton test, frequency (percentage)

According to the findings of the path analysis, the percentage change in the RANKL/OPG, TNF-α, and TGF-β levels before and after treatment was affected by the type of medication (*p* < 0.05). Age, gender, tooth region and smoking habit did not have any effect on pretreatment or posttreatment levels of RANKL/OPG, TNF-α, and TGF-β levels (*p* > 0.05). There was no statistically significant effect of any variable on the percentage change in PGE2 in both groups (*p* > 0.05) (Table 2).

**Table 2:** Analysis of the effect of independent variables on percentage changes

Dependent variables		Independent variables	*β*_1_	*β*_2_	Standard error	Test statistics	*p* value	R2
Percentage change of RANKL/OPG	< ---	Group (Reference: calcium silicate)	0.678	31.852	4.433	7.185	**< 0.001**	0.475
Percentage change of RANKL/OPG	< ---	Age	− 0.073	− 0.164	0.213	− 0.77	0.441	
Percentage change of RANKL/OPG	< ---	Gender (Reference: male)	− 0.031	− 1.478	5.016	− 0.295	0.768	
Percentage change of RANKL/OPG	< ---	Tooth region (Reference: mandibular tooth)	0.031	1.438	4.435	0.324	0.746	
Percentage change of RANKL/OPG	< ---	Smoking habit (Reference: no)	0.078	3.694	4.922	0.75	0.453	
Percentage change of PGE2	< ---	Group (Reference: calcium silicate)	0.219	5.683	3.485	1.631	0.109	0.168
Percentage change of PGE2	< ---	Age	0.169	0.212	0.149	1.424	0.155	
Percentage change of PGE2	< ---	Gender (Reference: male)	0.187	5.008	3.499	1.431	0.152	
Percentage change of PGE2	< ---	Tooth region (Reference: mandibular tooth)	0.192	5.007	3.094	1.618	0.106	
Percentage change of PGE2	< ---	Smoking habit (Reference: no)	0.137	3.597	3.433	1.048	0.295	
Percentage change of TGF-β	< ---	Group (Reference: calcium silicate)	0.713	32.869	4.097	8.023	**< 0.001**	0.534
Percentage change of TGF-β	< ---	Age	0.04	0.089	0.197	0.451	0.652	
Percentage change of TGF-β	< ---	Gender (Reference: male)	− 0.096	− 4.574	4.636	− 0.987	0.324	
Percentage change of TGF-β	< ---	Tooth region (Reference: mandibular tooth)	0.055	2.55	4.099	0.622	0.534	
Percentage change of TGF-β	< ---	Smoking habit (Reference: no)	0.074	3.459	4.549	0.76	0.447	
Percentage change of TNF-α	< ---	Group (Reference: calcium silicate)	0.257	6.735	3.049	2.209	**0.027**	0.199
Percentage change of TNF-α	< ---	Age	− 0.007	− 0.009	0.147	− 0.063	0.950	
Percentage change of TNF-α	< ---	Gender (Reference: male)	− 0.141	− 3.79	3.45	− 1.099	0.272	
Percentage change of TNF-α	< ---	Tooth region (Reference: mandibular tooth)	0.178	4.654	3.05	1.526	0.127	
Percentage change of TNF-α	< ---	Smoking habit (Reference: no)	0.233	6.142	3.385	1.815	0.070	

Values in bold indicate statistical significance at *p* < 0.05

*β*_*1*_ standardized beta coefficient, *β*_*2*_ unstandardized beta coefficient

According to the intragroup analysis, there was a statistically significant decrease in the RANKL/OPG, TNF-α, PGE-2 and TGF-β levels before and after treatment in both groups (*p* < 0.05) (Table 3). Intergroup analyses showed that there was a statistically significant differences among the groups in terms of the percentage change in the RANKL/OPG, TNF-α, and TGF-β levels before and after treatment (*p* < 0.05), with a greater percentage change observed in the calcium hydroxide-based root canal dressing. No statistically significant difference was observed in the percentage change of PGE2 between the groups (*p* > 0.05) (Table 4).

**Table 3:** Results of inter-group and intra-group comparisons

	Calcium silicate		Calcium hydroxide		Test statistics	*p* value
	Mean ± SD	Median (min.–max.)	Mean ± SD	Median (min.–max.)		
Pretreatemnt RANKL/OPG	0.60 ± 0.15	0.63 (0.27–0.81)	0.60 ± 0.13	0.57 (0.45–0.86)	420.500	0.663*
Posttreatment RANKL/OPG	0.51 ± 0.17	0.56 (0.19–0.79)	0.30 ± 0.10	0.27 (0.17–0.58)	165.000	**< 0**.001*
Test statistics	− 3.733		− 4.782			
*p* value**	**< 0**.**001**		**< 0**.**001**			
Pretreatemnt PGE2	201.24 ± 41.04	210.77 (110.88–298.35)	204.98 ± 52.09	187.02 (155.05–361.86)	394.000	0.408*
Posttreatment PGE2	179.88 ± 39.79	187.74 (97.57–254.13)	167.53 ± 32.57	165.52 (91.68–223.35)	352.000	0.147*
Test statistics	-4.618		-4.741			
*p* value**	** < 0**.**001**		** < 0**.**001**			
Pretreatemnt TGF-β	23.65 ± 7.84	25.78 (10.18–40.13)	44.45 ± 7.12	45.57 (30.91–59.12)	17.000	**< 0**.001*
Posttreatment TGF-β	18.45 ± 5.92	19.10 (9.26–29.45)	21.65 ± 6.13	23.24 (10.46–32.86)	313.000	**0**.043*
Test statistics	− 3.877		− 4.782			
*p* value**	**< 0**.**001**		**< 0**.**001**			
Pretreatemnt TNF-α	268.59 ± 51.07	281.11 (156.92–340.42)	229.53 ± 28.64	224.98 (176.21–288.59)	3.654	**0**.001***
Posttreatment TNF-α	235.65 ± 46.34	244.72 (143.78–319.13)	184.48 ± 31.32	193.89 (107.96–239.22)	5.011	**< 0**.001***
Test statistics	5.017		9.190			
*p* value****	**< 0**.**001**		**< 0**.**001**			

Values in bold indicate statistical significance at *p* < 0.05

Pre- and posttreatment receptor activator of nuclear factor kappa B ligand (RANKL)/osteoprotegerin (OPG), PGE2, TGF-β, and TNF-α levels (*SD* standard deviation)

*Mann–Whitney *U* test

**Wilcoxon test

***Independent two-sample *t*-test

****Paired two-sample *t*-test

**Table 4:** Comparison of percentage changes between groups

	Calcium silicate		Calcium hydroxide		Test statistics	p value
	Mean ± SD	Median (min–max)	Mean ± SD	Median (min–max)		
Percentage changes of RANKL/OPG	15.66 ± 19.45	12.50 (− 24.73–61.01)	48.38 ± 15.28	49.18 (16.27–78.40)	− 7.247	** < 0**.001*
Percentage changes of PGE2	10.29 ± 10.63	6.71 (− 2.29–51.41)	16.32 ± 14.72	11.30 (− 1.00–56.58)	547.000	0.152**
Percentage changes of TGF-β	18.03 ± 21.24	15.27 (− 18.66–63.34)	51.80 ± 9.32	48.93 (40.20–77.67)	816.000	** < 0**.001**
Percentage changes of TNF-α	11.37 ± 12.58	10.98 (− 17.95–43.00)	19.32 ± 12.86	20.11 (− 33.11–51.59)	659.000	**0**.002**

Values in bold indicate statistical significance at *p* < 0.05

*Independent two-sample *t*-test

**Mann–Whitney *U* test

Pre- and post-treatment RANKL/OPG, TNF-α, PGE2, and TGF-β levels and percentage changes in the calcium silicate and calcium hydroxide groups (*SD* standard deviation)

## Discussion

The immune responses during the formation of periapical lesions are characterized by the involvement of various cytokines and chemokines. Endotoxins and proinflammatory cytokines contribute to the development of periapical lesions. RANKL/OPG, TNF-α, PGE2, and TGF-β indicate the presence of bone resorption and remodeling in the periapical area [18]. In this randomized controlled clinical trial, the effects of tricalcium silicate-based and calcium hydroxide-based intracanal medicaments on the pre- and post-treatment levels of RANKL/OPG, TNF-α, PGE2, and TGF-β were evaluated in single-rooted teeth with periapical lesions requiring nonsurgical endodontic retreatment. A statistically significant difference in the levels before and after treatment was observed in both groups. Based on these results, the null hypothesis was rejected (Fig. 2).

In the present study, a statistically significant difference was observed in the RANKL/OPG ratio before and after treatment in the calcium hydroxide-based intracanal dressing group. Uluköylü et al. [17] showed that calcium hydroxide based intracanal dressing did not create a statistically significant difference in the RANKL/OPG levels before and after treatment. Different concentrations of calcium hydroxide may affect the release of RANKL and other mediators in the periapical region [19]. In addition, the concentration of calcium hydroxide may influence local tissue repair [20]. The inconsistency could be due to the differences in sample size, potential variations in dressing concentration, or factors related to the patient or clinician.

Insufficient antimicrobial activity may lead to a failure to quickly suppressing inflammation and result in the formation of an acidic environment, which is unfavorable for calcium salt precipitation [21, 22]. Calcium hydroxide- and calcium silicate-based root canal dressings promote mineralization and provide an alkaline environment that supports tissue repair due to their dissociation into calcium and hydroxyl ions [10, 23, 24]. This could have contributed to the statistically significant reduction in the reduced RANKL/OPG, TNF-α, and TGF-β levels after treatment. However, Bio-C Temp has a lower ability to release Ca^+2^ and OH^−^ and a lower antibacterial effect than calcium hydroxide based intracanal dressings [15]. Additionally, it has been demonstrated that Bio-C Temp causes slow calcium release during the first 24 h, resulting in lower pH values [25]. The higher percentage changes in RANKL/OPG, TNF-α, and TGF-β levels in the calcium hydroxide group can be explained by the higher pH environment provided by calcium hydroxide-based intracanal dressing.

Meng et al. [26] was evaluated the effect of calcium hydroxide on the expression of TNF-α and IL-6 in osteoblasts in the periapical region. The research findings revealed that calcium hydroxide reduced the levels of TNF-α and IL-6 levels in periapical osteoblasts. Another study showed that TNF-α was effectively denatured by calcium hydroxide [27]. The findings showed that the application of calcium hydroxide reduced TNF-α expression, which is consistent with the results of present study. Bio-C Temp had less antibacterial activity than the calcium hydroxide. The low antibacterial activity of Bio-C Temp could be attributed to fewer ions formed in the hydration reaction than the calcium hydroxide [15]. The higher percentage change in RANKL/OPG and TNF-α levels in the calcium hydroxide-based medicament group in the present study could be attributed to the release of lower calcium and hydroxyl ions from Bio-C Temp, which is related to its physicochemical properties.

Calcium hydroxide-based intracanal dressing contains an aqueous carrier, while the tricalcium silicate-based intracanal dressing has a viscous carrier-polyethylene glycol. According to the literature, viscous carriers dissolve less than aqueous carriers due to their higher molecular weight. [28]. While aqueous solutions like water and saline promote rapid ion release, viscous carriers like propylene glycol cause calcium and hydroxyl ions to be released into the environment over a longer period [29]. In the present study, the more effective reduction of cytokine levels by the calcium hydroxide-based intracanal dressing may be related to the properties of their vehicles.

Statistically significant reduction in PGE-2 levels was observed in both groups, with no difference in the percentage change between the groups. Karataş et al. [30] demonstrated a significant reduction in PGE-2 levels in teeth treated with a calcium hydroxide-based intracanal dressing. In addition, studies have reported a decrease in PGE-2 levels from the first to the second appointment [31, 32], which are consistent with our findings. While PGE-2 alone may not be responsible for the healing of apical lesions after endodontic procedures, a reduction in PGE-2 levels could be a factor that positively influences the healing of apical periodontitis. A previous clinical study reported a correlation between PGE-2 levels and pain [33]. PGE-2 levels in periapical fluids are related to clinical symptoms [34]. In the present study, no statistically significant difference was found in the percentage changes in the PGE-2 levels between the groups before and after treatment. This could be attributed to the inclusion of teeth with asymptomatic apical periodontitis in the study, which may have led to similar PGE-2 levels in both groups.

Ferreira et al. [35] evaluated the effects of irrigation solutions and calcium hydroxide based intracanal dressing on the release of TGF-β and vascular endothelial growth factor (VEGF) from cervical root dentin and found that calcium hydroxide as an intracanal dressing resulted in a higher release of TGF-β from cervical root dentin. A previous study showed that tricalcium silicate-based materials release growth factors, such as TGF-β, from dentin matrix binding sites, supporting the formation of reparative dentin [36]. In the present study, the statistically significant decrease in the initially high TGF-β levels by day 7 could be attributed to the reduction of inflammatory mediators in the environment, leading to a decrease in TGF-β levels as well.

There are studies indicating that there is no significant difference in the antibacterial efficacy of NaOCl at different concentrations. In a study by Siqueira et al. [37], it was found that the antibacterial effectiveness of 1% NaOCl was similar to that of 5% NaOCl. Dunavant et al. also stated that both 1% NaOCl and 6% NaOCl were efficient in eliminating E. faecalis biofilm [38]. Retana-Lobo et al. [39] noted that NaOCl affects dentin mechanical properties by degrading its organic components through the loss of DMP1-CT expression in the canal lumen, with this effect being more pronounced when 2.5% NaOCl was used. Based on these studies, 1% NaOCl was used to achieve effective chemo-mechanical preparation while minimizing potential damage to surrounding tissues.

Various opinions exist regarding the optimal duration for maintaining root canal dressing within the canal [40, 41]. Shuping et al. [42] reported a 92.5% reduction in bacteria after using calcium hydroxide in root canals for 1 week. A systematic review indicated that keeping calcium hydroxide for 7–45 days resulted in similar antimicrobial effects [43]. Martinho et al. [31] demonstrated that keeping a calcium hydroxide-based root canal dressing for 7 days provided effective antimicrobial activity, and there was no statistical difference in the reduction of IL-1β, TNF-α, and PGE2 levels between the 7-day and 14-day applications. Therefore, the root canal dressings were retained for a duration of 7 days.

The limitation of this study is the use of a premixed medicament containing 45% calcium hydroxide, rather than calcium hydroxide powder. Factors that determine the effectiveness of calcium hydroxide dressing include the preparation method of the paste, the level of radiopacity, and the application technique within the root canal [44]. Additionally, achieving a homogeneous placement of calcium hydroxide along the root canal is challenging. The consistency of the carriers combined with calcium hydroxide affects clinical ease of use and may hinder its uniform distribution within the canal system [45]. Consequently, variations in the application methods of the dressing may influence the results. Since the calcium silicate-based root canal dressing is in an injectable form, an injectable calcium hydroxide-based medicament was selected to ensure uniform application of the medicaments within the root canal.

## Conclusion

Within the limitations of this study, it can be concluded that the calcium silicate-based root canal dressing was effective in reducing RANKL/OPG, TNF-α, PGE-2, and TGF-β levels in apical periodontitis. However, it should be noted that the calcium hydroxide-based dressing resulted in a greater percentage reduction in RANKL/OPG, TNF-α, and TGF-β levels. The effects of both dressings on PGE-2 levels were similar. Further clinical studies are needed to investigate the effects of tricalcium silicate-based intracanal dressing on cytokines in periapical tissues.

## Data Availability

Not applicable.

## References

[1] Tibúrcio-Machado CS, Michelon C, Zanatta FB, et al. The global prevalence of apical periodontitis: a systematic review and meta-analysis. Int Endod J. 2021;54:712–35. 10.1111/IEJ.13467. 33378579 10.1111/iej.13467

[2] Nair PNR. On the causes of persistent apical periodontitis: a review. Int Endod J. 2006;39:249–81. 10.1111/J.1365-2591.2006.01099.X. 16584489 10.1111/j.1365-2591.2006.01099.x

[3] Ye L, Cao L, Song W, et al. Interaction between apical periodontitis and systemic disease (review). Int J Mol Med. 2023. 10.3892/IJMM.2023.5263. 37264964 10.3892/ijmm.2023.5263 PMC10249585

[4] Abbott PV. Classification, diagnosis and clinical manifestations of apical periodontitis. Endod Top. 2004;8(1):36–54. 10.1111/j.1601-1546.2004.00098.x.

[5] Hofbauer LC. Osteoprotegerin ligand and osteoprotegerin: novel implications for osteoclast biology and bone metabolism. Eur J Endocrinol. 1999;141:195–210. 10.1530/EJE.0.1410195. 10474114 10.1530/eje.0.1410195

[6] Tani-Ishii N, Wang C-Y, Stashenko P. Immunolocalization of bone-resorptive cytokines in rat pulp and periapical lesions following surgical pulp exposure. Oral Microbiol Immunol. 1995;10:213–9. 10.1111/J.1399-302X.1995.TB00145.X. 8602333 10.1111/j.1399-302x.1995.tb00145.x

[7] Abbott PV. Medicaments: aids to success in endodontics. Part 1. A review of the literature. Aust Dent J. 1990;35:438–48. 10.1111/J.1834-7819.1990.TB05427.X. 2073192 10.1111/j.1834-7819.1990.tb05427.x

[8] Siqueira JF, De Uzeda M. Disinfection by calcium hydroxide pastes of dentinal tubules infected with two obligate and one facultative anaerobic bacteria. J Endod. 1996;22:674–6. 10.1016/S0099-2399(96)80062-8. 9220753 10.1016/S0099-2399(96)80062-8

[9] Siqueira JF, Lopes HP. Mechanisms of antimicrobial activity of calcium hydroxide: a critical review. Int Endod J. 1999;32:361–9. 10.1046/J.1365-2591.1999.00275.X. 10551109 10.1046/j.1365-2591.1999.00275.x

[10] Mizuno M, Banzai Y. Calcium ion release from calcium hydroxide stimulated fibronectin gene expression in dental pulp cells and the differentiation of dental pulp cells to mineralized tissue forming cells by fibronectin. Int Endod J. 2008;41:933–8. 10.1111/J.1365-2591.2008.01420.X. 19133082 10.1111/j.1365-2591.2008.01420.x

[11] Kahler SL, Shetty S, Andreasen FM, Kahler B. The effect of long-term dressing with calcium hydroxide on the fracture susceptibility of teeth. J Endod. 2018;44:464–9. 10.1016/J.JOEN.2017.09.018. 29254817 10.1016/j.joen.2017.09.018

[12] López-García S, Pecci-Lloret MR, Guerrero-Gironés J, et al. Comparative cytocompatibility and mineralization potential of bio-c sealer and TotalFill BC sealer. Mater (Basel, Switzerland). 2019. 10.3390/MA12193087. 10.3390/ma12193087 PMC6804055 31546696

[13] Giacomino CM, Wealleans JA, Kuhn N, Diogenes A. Comparative biocompatibility and osteogenic potential of two bioceramic sealers. J Endod. 2019;45:51–6. 10.1016/J.JOEN.2018.08.007. 30558798 10.1016/j.joen.2018.08.007

[14] de Campos IVB, Vieira WA, de Almeida RF, et al. In vitro dental discoloration provoked by intracanal calcium silicate-based dressing used for regenerative endodontic procedures: an one-year spectrometric analysis. J Endod. 2023;49:846–51. 10.1016/J.JOEN.2023.04.009. 37121270 10.1016/j.joen.2023.04.009

[15] Guerreiro JCM, Ochoa-Rodrígez VM, Rodrigues EM, et al. Antibacterial activity, cytocompatibility and effect of Bio-C Temp bioceramic intracanal medicament on osteoblast biology. Int Endod J. 2021;54:1155–65. 10.1111/IEJ.13502. 33638900 10.1111/iej.13502

[16] Lopes CS, Delfino MM, Tanomaru-Filho M, et al. Bioactive potential of Bio-C Temp demonstrated by systemic mineralization markers and immunoexpression of bone proteins in the rat connective tissue. J Mater Sci Mater Med. 2024. 10.1007/S10856-024-06781-3. 38353838 10.1007/s10856-024-06781-3 PMC10867037

[17] Uluköylü E, Karataş E, Albayrak M, Bayır Y. Effect of calcium hydroxide alone or in combination with ibuprofen and ciprofloxacin on nuclear factor kappa b ligand and osteoprotegerin level in periapical lesions: a randomized controlled clinical study. J Endod. 2019;45:1489–95. 10.1016/J.JOEN.2019.09.011. 31706622 10.1016/j.joen.2019.09.011

[18] Marçal JRB, Samuel RO, Fernandes D, et al. T-helper cell type 17/regulatory T-cell immunoregulatory balance in human radicular cysts and periapical granulomas. J Endod. 2010;36:995–9. 10.1016/J.JOEN.2010.03.020. 20478453 10.1016/j.joen.2010.03.020

[19] Han B, Wang X, Liu J, et al. Influence of calcium hydroxide-loaded microcapsules on osteoprotegerin and receptor activator of nuclear factor kappa B ligand activity. J Endod. 2014;40:1977–82. 10.1016/J.JOEN.2014.08.008. 25266469 10.1016/j.joen.2014.08.008

[20] Silva EJNL, Herrera DR, Almeida JFA, et al. Evaluation of cytotoxicity and up-regulation of gelatinases in fibroblast cells by three root repair materials. Int Endod J. 2012;45:815–20. 10.1111/J.1365-2591.2012.02038.X. 22452531 10.1111/j.1365-2591.2012.02038.x

[21] Nekoofar MH, Namazikhah MS, Sheykhrezae MS, et al. pH of pus collected from periapical abscesses. Int Endod J. 2009;42:534–8. 10.1111/J.1365-2591.2009.01550.X. 19460003 10.1111/j.1365-2591.2009.01550.x

[22] Lu X, Leng Y. Theoretical analysis of calcium phosphate precipitation in simulated body fluid. Biomaterials. 2005;26:1097–108. 10.1016/J.BIOMATERIALS.2004.05.034. 15451629 10.1016/j.biomaterials.2004.05.034

[23] Estrela C, Pécora JD, Souza-Neto MD, Estrela CR, Bammann LL. Effect of vehicle on antimicrobial properties of calcium hydroxide pastes. Braz Dent J. 1999;10(2):63–72. 10863391

[24] Villa N, Dos Santos VV, da Costa UM, et al. A new calcium silicate-based root canal dressing: physical and chemical properties, cytotoxicity and dentinal tubule penetration. Braz Dent J. 2020;31:598–604. 10.1590/0103-6440202003376. 33237230 10.1590/0103-6440202003376

[25] Capitanio BL, Hashizume LN, Kuga MC, et al. Analysis of pH, calcium ion release, and energy dispersive spectroscopy of a bioceramic root canal dressing. Braz Dent J. 2023;34:54–61. 10.1590/0103-6440202305506. 37909642 10.1590/0103-6440202305506 PMC10642275

[26] Meng LJ, Qiu LH, Yu YQ, Zhan FL, Zhang L, Zhang XF. The effect of calcium hydroxide on IL-6 and TNF-α expression of osteoblast in periapical tissues. Shanghai J Stomatol. 2016;25:32–7. 27063305

[27] Khan AA, Sun X, Hargreaves KM. Effect of calcium hydroxide on proinflammatory cytokines and neuropeptides. J Endod. 2008;34:1360–3. 10.1016/J.JOEN.2008.08.020. 18928847 10.1016/j.joen.2008.08.020 PMC4524651

[28] Fava LRG, Saunders WP. Calcium hydroxide pastes: classification and clinical indications. Int Endod J. 1999;32:257–82. 10.1046/J.1365-2591.1999.00232.X. 10551118 10.1046/j.1365-2591.1999.00232.x

[29] Gomes BPFDA, Ferraz CCR, Vianna ME, et al. In vitro antimicrobial activity of calcium hydroxide pastes and their vehicles against selected microorganisms. Braz Dent J. 2002;13:155–61. 10.1590/S0103-64402002000300002. 12428587 10.1590/s0103-64402002000300002

[30] Karataş E, Uluköylü E, Albayrak M, Bayır Y. Effect of calcium hydroxide alone or in combination with ibuprofen and ciprofloxacin on postoperative pain and periapical prostaglandin E2 level: a randomized clinical study. Prostaglandins Other Lipid Mediat. 2021;153: 106525. 10.1016/J.PROSTAGLANDINS.2020.106525. 33383182 10.1016/j.prostaglandins.2020.106525

[31] Martinho FC, Gomes CC, Nascimento GG, et al. Clinical comparison of the effectiveness of 7- and 14-day intracanal medications in root canal disinfection and inflammatory cytokines. Clin Oral Investig. 2018;22:523–30. 10.1007/S00784-017-2143-X. 28589472 10.1007/s00784-017-2143-x

[32] Shimauchi H, Takayama SI, Miki Y, Okada H. The change of periapical exudate prostaglandin E2 levels during root canal treatment. J Endod. 1997;23:755–8. 10.1016/S0099-2399(97)80350-0. 9487853 10.1016/s0099-2399(97)80350-0

[33] Grga D, Dželetović B, Damjanov M, Hajdukovic-Dragojlovic L. Prostaglandin E2 in apical tissue fluid and postoperative pain in intact and teeth with large restorations in two endodontic treatment visits. Srp Arh Celok Lek. 2013;141:17–21. 10.2298/SARH1302017G. 23539905 10.2298/sarh1302017g

[34] Takayama SI, Miki Y, Shimauchi H, Okada H. Relationship between prostaglandin E2 concentrations in periapical exudates from root canals and clinical findings of periapical periodontitis. J Endod. 1996;22:677–80. 10.1016/S0099-2399(96)80063-X. 9220754 10.1016/s0099-2399(96)80063-x

[35] Ferreira LN, Puppin-Rontani RM, Pascon FM. Effect of intracanal medicaments and irrigants on the release of transforming growth factor beta 1 and vascular endothelial growth factor from cervical root dentin. J Endod. 2020;46:1616–22. 10.1016/J.JOEN.2020.07.034. 32795548 10.1016/j.joen.2020.07.034

[36] Da Pedrosa MS, Nogueira FN, Sipert CR. Calcium silicate-based cements affect the cell viability and the release of TGF-β1 from apical papilla cells. Braz Dent J. 2021;32:1–7. 10.1590/0103-6440202104598. 35019013 10.1590/0103-6440202104598

[37] Siqueira JF, Rôças IN, Favieri A, Lima KC. Chemomechanical reduction of the bacterial population in the root canal after instrumentation and irrigation with 1%, 2.5%, and 5.25% sodium hypochlorite. J Endod. 2000;26:331–4. 10.1097/00004770-200006000-00006. 11199749 10.1097/00004770-200006000-00006

[38] Dunavant TR, Regan JD, Glickman GN, et al. Comparative evaluation of endodontic irrigants against *Enterococcus **faecalis* biofilms. J Endod. 2006;32:527–31. 10.1016/J.JOEN.2005.09.001. 16728243 10.1016/j.joen.2005.09.001

[39] Retana-Lobo C, Ramírez-Mora T, Murillo-Gómez F, et al. Final irrigation protocols affect radicular dentin DMP1-CT expression, microhardness, and biochemical composition. Clin Oral Investig. 2022;26:5491–501. 10.1007/S00784-022-04516-8. 35499657 10.1007/s00784-022-04516-8

[40] Vera J, Siqueira JF, Ricucci D, et al. One- versus two-visit endodontic treatment of teeth with apical periodontitis: a histobacteriologic study. J Endod. 2012;38:1040–52. 10.1016/J.JOEN.2012.04.010. 22794203 10.1016/j.joen.2012.04.010

[41] Tavares WLF, De Brito LCN, Henriques LCF, et al. Effects of calcium hydroxide on cytokine expression in endodontic infections. J Endod. 2012;38:1368–71. 10.1016/J.JOEN.2012.06.036. 22980179 10.1016/j.joen.2012.06.036

[42] Shuping GB, Ørstavik D, Sigurdsson A, Trope M. Reduction of intracanal bacteria using nickel-titanium rotary instrumentation and various medications. J Endod. 2000;26:751–5. 10.1097/00004770-200012000-00022. 11471648 10.1097/00004770-200012000-00022

[43] Sharma G, Ahmed HMA, Zilm PS, Rossi-Fedele G. Antimicrobial properties of calcium hydroxide dressing when used for long-term application: a systematic review. Aust Endod J. 2018;44:60–5. 10.1111/AEJ.12216. 29168274 10.1111/aej.12216

[44] Deveaux E, Dufour D, Boniface B. Five methods of calcium hydroxide intracanal placement: an in vitro evaluation. Oral Surg Oral Med Oral Pathol Oral Radiol Endod. 2000;89:349–55. 10.1016/S1079-2104(00)70101-6. 10710462 10.1016/s1079-2104(00)70101-6

[45] Grover C, Shetty N. Evaluation of calcium ion release and change in pH on combining calcium hydroxide with different vehicles. Contemp Clin Dent. 2014;5:434–9. 10.4103/0976-237X.142803. 25395755 10.4103/0976-237X.142803 PMC4229748

